# GATA2 haploinsufficient patients lack innate lymphoid cells that arise after hematopoietic cell transplantation

**DOI:** 10.3389/fimmu.2022.1020590

**Published:** 2022-10-03

**Authors:** Y. F. van Lier, L. Krabbendam, N. J. E. Haverkate, S. S. Zeerleder, C. E. Rutten, B. Blom, H. Spits, M. D. Hazenberg

**Affiliations:** ^1^ Department of Hematology, Amsterdam University Medical Centers (UMC), University of Amsterdam, Amsterdam, Netherlands; ^2^ Department of Experimental Immunology, Amsterdam Institute for Infection and Immunity Institute (AII), Cancer Center Amsterdam, Amsterdam University Medical Centers (UMC) location Academic Medical Center (AMC), Amsterdam, Netherlands; ^3^ Department of Hematology, Luzerner Kantonsspital, and University of Bern, Lucerne, Switzerland; ^4^ Department of Hematopoiesis, Sanquin Research, Amsterdam, Netherlands

**Keywords:** innate lymphocyte cells (ILCs), NK cells, GATA 2, allogeneic haematopoietic cell transplantation, reconstitution, MonoMAC syndrome

## Abstract

Innate lymphoid cells (ILC) are important barrier tissue immune regulators. They play a pivotal role in early non-specific protection against infiltrating pathogens, regulation of epithelial integrity, suppression of pro-inflammatory immune responses and shaping the intestinal microbiota. GATA2 haploinsufficiency causes an immune disorder that is characterized by bone marrow failure and (near) absence of monocytes, dendritic cells, B cells and natural killer (NK) cells. T cells develop normally, albeit at lower numbers. Here, we describe the absence of ILCs and their progenitors in blood and bone marrow of two patients with GATA2 haploinsufficiency and show that all subsets of ILCs appear after allogeneic hematopoietic stem cell transplantation, irrespective of the preparative conditioning regimen. Our data indicate that GATA2 is involved in the development of hematopoietic precursor cells (HPC) towards the ILC lineage.

## Highlights

1. Patients with GATA2 haploinsufficiency lack ILCs in blood and ILC progenitors in bone marrow.
2. Replacement of GATA2 deficient hematopoiesis by donor hematopoiesis is associated with the emergence of all ILC subsets, irrespective of pre-transplant conditioning regimen.

## Introduction

The transcription factor Guanine Adenine Thymine Adenine binding protein 2 (GATA2) is required for hematopoietic stem cell development and maintenance (1–3). GATA2 mutations reduce its expression and induce haploinsufficiency. The resulting immune disorder is characterized by the (near) absence of monocytes, dendritic cells (DC), B cells and natural killer (NK) cells (4, 5), which can manifest with recurrent, potentially life-threatening infections, and myeloid malignancies, e.g. myelodysplastic syndrome and acute myeloid leukemia (AML) (6–8). Innate lymphoid cells (ILCs) play pivotal roles in the early, non-specific protection against infiltrating pathogens, regulation of epithelial integrity and maintenance of tissue homeostasis through antigen-independent cytokine production (9, 10). It is currently not known whether a genetic GATA2 defect results in loss of ILCs and whether this contributes to the clinical phenotype of GATA2 haploinsufficiency.

ILC deficiency has been described in patients with severe combined immune deficiency (SCID), who also lack T cells and NK cells and have functionally impaired B cells (11–13). The majority of SCID patients undergo an allogeneic hematopoietic cell transplantation (HCT) to correct their immune system. Following non-myeloablative conditioning chemotherapy, ILCs did not recover post-transplant in these patients (11). These patients did not obtain full donor chimerism after HCT, as only the lymphoid lineage was replaced by donor hematopoiesis while the other leukocyte lineages remained of recipients’ origin. A study from our group in AML patients demonstrated ILC depletion by pre-transplantation conditioning radio- and chemotherapy and variable ILC reconstitution post-transplantation, irrespective of the preparative chemotherapy regimen intensity (i.e. myeloablative or non-myeloablative) (14). These patients obtained full donor chimerism post-transplantation. It remains to be determined which intrinsic or extrinsic factors are essential for successful ILC reconstitution.

Here, we examined the presence of ILCs and their precursors in bone marrow and blood of two patients with GATA2 haploinsufficiency. Both patients required an allogeneic HCT, offering an opportunity to investigate the emergence of ILCs after myeloablative (MA) and reduced intensity (RIST) preparative regimens in patients with congenital ILC deficiency.

## Methods

### Patients

**Figure 1:**
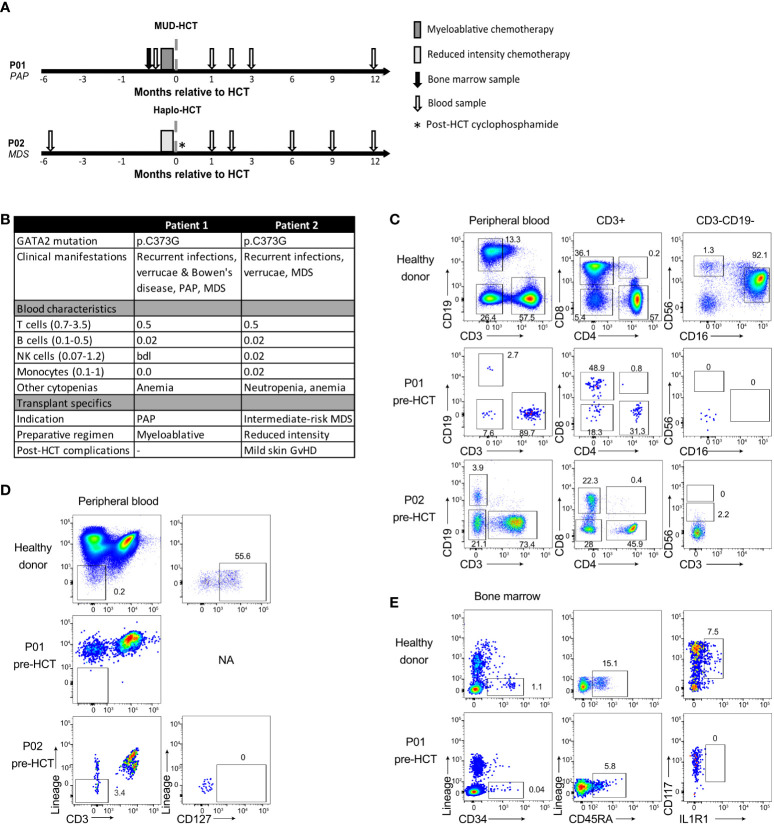
Deficiency of T, B, NK cell and ILC in GATA2 haploinsufficiency. **(A)** Schematic overview of patient treatment schedules and timing of sample collection. Conditioning regimens: P01: busulfan, fludarabine, anti-thymocyte globulin; P02: cyclophosphamide, fludarabine, total body irradiation (TBI, 2 Gy) and post-transplant cyclophosphamide. Haplo, haploidentical; HCT, hematopoietic cell transplantation; MDS, myelodysplastic syndrome; MUD, matched unrelated donor; PAP, pulmonary alveolar proteinosis. **(B)** Patient characteristics and absolute cell numbers (x109/L) of T cells, B cells, NK cells and monocytes (Mono) in peripheral blood at time of diagnosis of GATA2 haploinsufficiency or shortly after. bdl, below detection level; GvHD, graft-versus-host disease; HCT, hematopoietic cell transplantation; NA, not applicable; MDS, myelodysplastic syndrome; PAP, pulmonary alveolar proteinosis. **(C)** Flow cytometric analysis of T cells, B cells and NK cells in peripheral blood cryopreserved mononuclear cells from a healthy donor, P01 and P02 collected prior to allogeneic HCT. Cells are pre-gated on viable CD45+ lymphocytes (**Figure S1**) and B cells are CD19+, T cells are CD3+ and categorized into CD4+, CD8+, CD4-CD8- and CD4+CD8+ T cells. NK cells are CD19-CD3- cells and are categorized into CD56brightCD16- NK cells and CD56dimCD16+ NK cells. For P02, no CD16 staining was available. Full gating strategy is displayed in **Figure S1A–C**. **(D)** Flow cytometric analysis and gating strategy used for identification of the CD127+ ILC population in peripheral blood cryopreserved mononuclear cells from healthy donors (representative of n=3) and patients. Cells are pre-gated on viable CD45+ (**Figure S2A–C**) and ILCs are identified as lineage (CD1a, CD3, CD4, CD5, CD14, CD16, CD19, CD34, CD94 CD123, BDCA2, TCRαβ, TCRγδ and FcER1α)- CD3- CD127+ cells. NA = not applicable. Full gating strategy is displayed in **Figure S2A–C**). **(E)** Flow cytometric analysis and gating strategy used for identification of the multipotent hematopoietic progenitor cells (HPC) and ILC precursors in cryopreserved mononuclear cells from BM of a healthy donor and P01. HPC are selected as Lineage- (CD1a, CD3, CD4, CD5, CD14, CD16, CD19, CD94 CD123, BDCA2, TCRαβ, TCRγδ and FcER1α) CD34+CD45RA+ and within this population ILC precursors are selected as CD117+IL1R1+. P02 had too few HPC to staining for HPC and ILC precursors. Full gating strategy is displayed in **Figure S2D**.

Blood and bone marrow (BM) samples were obtained from GATA2 haploinsufficient patients that underwent allogeneic HCT at Amsterdam UMC, Amsterdam, The Netherlands. Samples were obtained prior to HCT and at consecutive time points up to 1 year after HCT (**Figure 1A**). Healthy donor buffy coats were provided by the Dutch blood bank (Sanquin, Amsterdam). All participants provided informed consent. The study was approved by the Institutional Review Board, in accordance with the Declaration of Helsinki.

### Immunophenotyping

Peripheral blood mononuclear cells (PBMC) and BM mononuclear cells (BMMC) were isolated by Ficoll-Hypaque density gradient (Lymphoprep; Axis-Shield). BMMCs were directly analyzed following isolation; PBMCs were cryopreserved in RPMI 1640 (Gibco) + 10% fetal calf serum +10% DMSO in liquid nitrogen. PBMCs were thawed, washed with PBS and stained for 30 min at 4°C with fluorochrome-conjugated antibodies (**Table S1**). Cell suspensions were measured by flow cytometry using a LSR Fortessa (BD Biosciences) and analyzed using FlowJo software (FlowJo LLC, Ashland, OR).

## Results

### Patient characteristics

Family members patient 1 (P01) and patient 2 (P02) carried GATA2 missense mutation p.C373G (**Figure 1B**). It is likely that this mutation strongly affects the transcriptional activity of GATA2 since it was previously observed that a mutation at the same position, p.C373R, results in a disordered GATA2 protein with reduced transcriptional activity and DNA binding capacity (7). Both patients displayed features of GATA2 haploinsufficiency including recurrent infections, verrucae and bone marrow dysplasia. A detailed overview of the clinical manifestations of both patients has been described previously (15).

The peripheral blood of both patients showed the typical (near) absence of monocytes, B cells and NK cells (**Figure 1C**, **Figure S1**) and low T cell numbers compared to healthy individuals. The T cell pool contained a relatively high percentage of CD4- CD8- T cells (**Figures 1C**, **Figure S1**).

### GATA2 haploinsufficient patients are ILC deficient

The ILC composition was investigated by gating for CD45+ lineage- CD127+ lymphocytes (**Figures S2A–C**). In contrast to healthy individuals, peripheral blood ILCs were absent in both P01 and P02 (**Figure 1D**). To examine whether the absence of peripheral ILCs was caused by a developmental defect, we analyzed the presence of ILC progenitors (ILCp) in the bone marrow of P01. Bone marrow ILCp’s are enclosed within lineage^-^ CD34^+^ CD45RA^+^ multipotent hematopoietic progenitor cells (HPC) and express CD117 and IL1R1 (16). P01 displayed a low frequency of HPCs in the bone marrow compared to healthy individuals, and CD117+IL1R1+ ILCp’s were completely absent (**Figure 1E**, **Figure S2D**).

### Reconstitution of T, B and NK cells after allogeneic HCT

**Figure 2:**
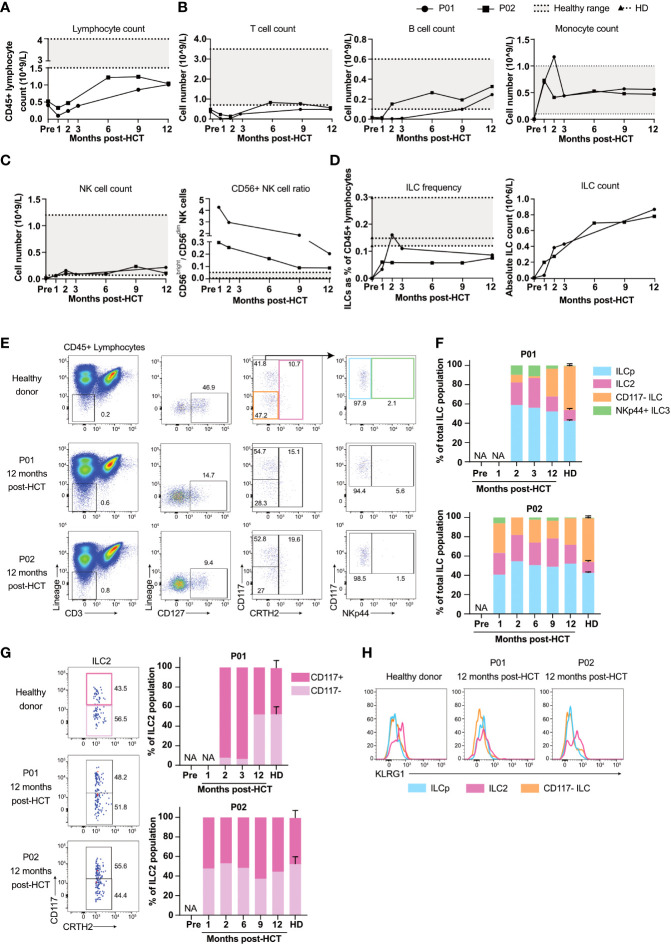
Reconstitution of T, B, NK cells and ILCs after allogeneic HCT. **(A)** Peripheral blood CD45+ lymphocyte number of P01 and P02 pre-transplantation and at consecutive timepoints post-transplantation. **(B)** Panels show the absolute count of peripheral blood T cells and B cells, in P01 and P02 calculated with the obtained frequencies from our flow cytometry data (**Figure S1**) and the absolute CD45+ lymphocyte numbers from **(A)**. Numbers of monocytes are obtained from the clinical lab. The grey area in the panels indicate the healthy range as employed by the clinical laboratory. **(C)** Panels show the absolute count of peripheral blood NK cells (left panel) calculated with the obtained frequencies from our flow cytometry data (**Figure S1**) and the absolute CD45+ lymphocyte numbers from **(A)**, and the ratio of CD56^dim^CD16+ and CD56^bright^CD16- (right panel). The dashed lines indicate the individual healthy donor (HD) values with the grey area showing the range of the HD values. **(D)** ILC frequencies in peripheral blood pre- and post-transplantation calculated as the percentage of viable CD45+ lymphocytes (upper panel) and absolute numbers (lower panel) of P01, P02 and 3 healthy donors (HD; dashed lines indicate the individual HD values with the grey area showing the range of the HD values). The absolute number of peripheral blood ILCs in P01 and P02 were calculated with the frequencies obtained from our flow cytometry data (**Figure S2A–C**) and the absolute CD45+ lymphocyte numbers from**(A)**. **(E)** Flow cytometric analysis of the cryopreserved PBMC from a healthy donor (representative for n=3) and P01 and P02 collected at 12 months post-transplantation. ILCs are selected as viable CD45+ Lin- CD3- CD127+ cells. CD117- ILC1s are gated for by selecting CD117- CRTH2- cells (orange gate), ILC2s are CRTH2+ (red gate) and CD117+CRTH2- cells (blue gate) can be subdivided into NKp44- ILCp or NKp44+ ILC3 (green gate). **(F)** Peripheral blood ILC subset frequencies within the CD127+ ILC population of healthy donors (N=3), P01 and P02. Samples were collected at indicated timepoints. NA = not applicable due to low cell numbers. **(G)** Quantification of CD117- and CD117+ ILC2s as a percentage of total CRTH2+ ILCs, gated for as in **(E)**. **(H)** Expression of KLRG1 on CD117- ILCs, ILCp and ILC2s, gated for as in **(E)**.

P01 received a myeloablative allogeneic HCT due to progressive lung dysfunction caused by pulmonary alveolar proteinosis (PAP), a well-described GATA2 deficiency-associated complication caused by dysfunctional alveolar macrophages (5, 15, 17, 18). P02 was diagnosed with intermediate-risk myelodysplastic syndrome and underwent reduced-intensity allogeneic HCT. An overview of the patients’ treatment schedules and sample collections is shown in **Figure 1A**. Routine short tandem repeat analyses post-transplantation revealed that both patients obtained full donor chimerism. In the 12 months follow-up post-transplant the number of lymphocytes gradually increased (**Figure 2A**), with the emergence of T cells, B cells, monocytes (**Figure 2B**) and CD56^bright^ and CD56^dim^ NK cells (**Figure 2C**) that reached levels comparable to healthy individuals (gating strategies in **Figure S1**). With respect to the NK cells, we observed that the ratio of CD56^bright^ to CD56^dim^ CD16^+^ NK cells gradually decreased suggesting that CD56^bright^ cells emerge more rapidly after transplantation than CD56^dim^ CD16^+^ NK cells (**Figure 2C** **)**.

### Establishment of the ILC pool following allogeneic HCT

The percentage of ILCs increased within two months after transplantation (**Figure 2D**, gating strategy in **Figures S2B, C**) but remained below normal values, consistent with previous literature (14, 19). Absolute ILC numbers increased from 0 pre-transplantation to ~0.8-0.9x10 (6) cells/liter blood within 12 months after allogeneic HCT (**Figure 2D**). The ILC population consisted of CD117^-^CRTH2^-^ cells that include ILC1s (**Figure 2E**, orange gate) (20, 21), CRTH2^+^ ILC2s (pink gate) (22), CD117^+^CRTH2^-^ ILCs that include ILCps (blue gate) (23, 24) and NKp44^+^ ILC3s (green gate (25). In healthy individuals, NKp44^+^ ILC3s are contained within tissues and hardly detectable in peripheral blood. Early after transplantation however, NKp44^+^ ILC3s were clearly present in the peripheral blood of both patients, which is in line with previous studies (**Figure 2E**, **Figures S2B, C** **)** (14). Twelve months post-transplantation the proportions of peripheral blood NKp44+ ILC3 of both patients were decreased and comparable to that of healthy individuals (**Figure 2F**).

The limited number of reconstituted ILCs prohibited us to perform functional assays, such as measuring cytokine production upon stimulation. Therefore we used an indirect way to obtain an understanding of the functional potential of the reconstituted ILCs by analyzing their expression of KLRG1 and the presence of CD117- and CD117+ ILC2s. We have previously reported that immature ILC2s differentiate from CD117+KLRG1+CRTH2- pILC2 *via* a CD117+KLRG1+CRTH2+ immature ILC2 to CD117-CRTH2+ ILC2 (24, 26). Moreover the CD117-CRTH2+ ILC2 are more polarized than the CD117+ILC2s as the latter cells express some RORγt and are capable of producing IL-17 (26). The presence of CD117-CRTH2+ ILC2 in the peripheral blood of the reconstituted GATA2 haploinsufficient patients would support the notion that the ILC2 are mature and fully capable of producing type 2 cytokines. The emergence of CD117- ILC2s was slow in P01, but at 12 months post-HCT their frequency was comparable to that of healthy donors. In P02, CD117- and CD117+ ILC2s recovered to a ratio comparable to healthy donors as early as one month post-HCT (**Figure 2G**). Expression of KLRG1 on ILC2s is associated with the potential of ILC2s to produce IL-10 (27). KLRG1 expression in P02 was similar to healthy donors, whereas KLRG1 expression was absent in P01 (**Figure 2H**). The absence of KLRG1 expression and the slow reconstitution of CD117- ILC2s in P01 suggest that ILC2 maturation, and potentially recovery of function, was slower in P01 than in P02.

## Discussion

This study shows that GATA2 haploinsufficiency is associated with a lack of ILC progenitors in the bone marrow and absence of ILCs and NK cells in the circulation, indicating a critical role for GATA2 in the development of NK cells and helper ILCs. Whereas T cells do develop from HPC in the absence of functional GATA2, our data suggest that ILC development is blocked in the transition from HPC towards ILCp, indicating that GATA2 is involved in the differentiation from HPC towards ILCp.

ILC reconstitution in the peripheral blood of both transplanted patients was slow compared to other innate effector cells such as monocytes and CD56+ NK cells. CD56^dim^CD16+ NK reconstituted slower than CD56^bright^CD16^-^ NK cells, which is consistent with the hypothesis that CD56^bright^ CD16- NK cells are precursors of CD56^dim^CD16+ NK cells. The delayed reconstitution of CD56^dim^CD16+ NK cells and ILCs over a period of 12 months more closely resembled the reconstitution dynamics of T and B lymphocytes. These data are in line with a previous study from our group among patients receiving allogeneic HCT for AML, in which we observed similar slow ILC recovery over time (14).

Reconstituting lymphocytes originate from peripheral expansion of mature cells present in the graft and/or from *de novo* development from donor-derived CD34+ hematopoietic stem cells. In a previous study the relative ILC content of the graft tended to correlate with ILC reconstitution after allogeneic HCT (28), raising the possibility that ILC reconstitution at least partly relies on expansion of mature ILCs in the blood. In the present study, we did not determine the proportion of mature ILCs in the grafts. Another limitation of this study is the lack of data on tissue-resident ILCs. This would provide insight in the capacity of reconstituted peripheral blood ILCs to migrate to tissues, where they exert their effector functions.

We here confirm our previous observation that ILC reconstitution occurs irrespective of pre-transplantation conditioning regimens (14). In SCID patients, ILC reconstitution was only observed in individuals who received a myeloablative allogeneic HCT after which the recipients’ hematopoiesis was fully replaced by donor hematopoiesis (11). ILCs and NK cells did not develop, however, when the non-T/ILC/NK leukocytes remained of recipients’ origin. Thus, complete donor chimerism of both lymphoid and myeloid hematopoietic lineages seems important for ILC reconstitution. In the present study, recipient hematopoiesis was fully replaced by donor hematopoiesis and the ILC pool, including NK cells, recovered in both patients, along with monocytes and B cells. Taken together, our data suggest that GATA2 is required for ILC development, and that ILC development following HCT only occurs when both the lymphoid and myeloid leukocyte lineages are replaced.

## Data availability statement

The original contributions presented in the study are included in the article/**Supplementary Material**. Further inquiries can be directed to the corresponding author.

## Ethics statement

The studies involving human participants were reviewed was approved by the Institutional Review Board at the the Amsterdam UMC, Academic Medical Center, Amsterdam, the Netherlands, in accordance with the Declaration of Helsinki. The patients/participants provided their written informed consent to participate in this study.

## Author contributions

Conceptualization: YV, LK, SZ, CR, BB, MH, HS; Investigation: YV, LK, NH; Visualization: YV, LK; Resources: MH; Funding acquisition: MH, HS; Project administration: YV, LK, MH; Supervision: MH, HS; Writing: YV, LK, HS, MH. All authors contributed to the article and approved the submitted version.

## Funding

This work was supported by a VIDI grant (NWO ZonMW #91715362) and a LSBR Fellowship (1438F) granted to MH and an advanced ERC grant (341038) granted to HS.

## Acknowledgments

We thank B. Hooibrink, T.M.M. van Capel and K.I.M Brandwijk for help with flow cytometry.

## Conflict of interest

The authors declare that the research was conducted in the absence of any commercial or financial relationships that could be construed as a potential conflict of interest.

## Publisher’s note

All claims expressed in this article are solely those of the authors and do not necessarily represent those of their affiliated organizations, or those of the publisher, the editors and the reviewers. Any product that may be evaluated in this article, or claim that may be made by its manufacturer, is not guaranteed or endorsed by the publisher.
