# Supplementary material for: GATA2 haploinsufficient patients lack innate lymphoid cells that arise after hematopoietic cell transplantation
Source: Front Immunol. 2022 Oct 3;13:1020590. doi: 10.3389/fimmu.2022.1020590 (PMC9577555; doi:10.3389/fimmu.2022.1020590)
Supplement: Supplementary file 1 [file DataSheet_1.pdf]

Supplemental figure 1

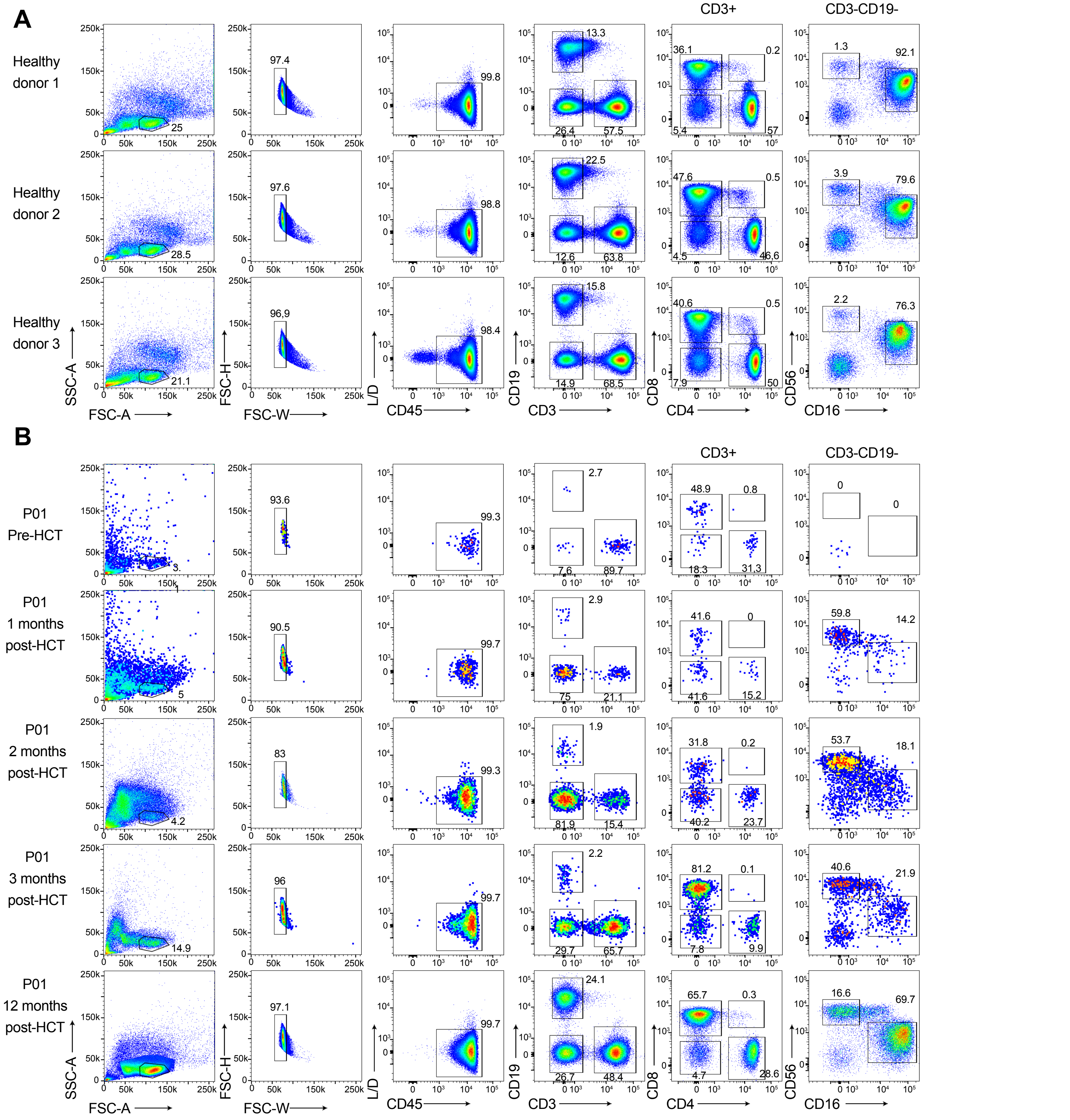

Figure S1. Flow cytometric analysis and gating strategy used for identification of T cells, B cells and NK cells in peripheral blood

Supplemental figure 1 continued

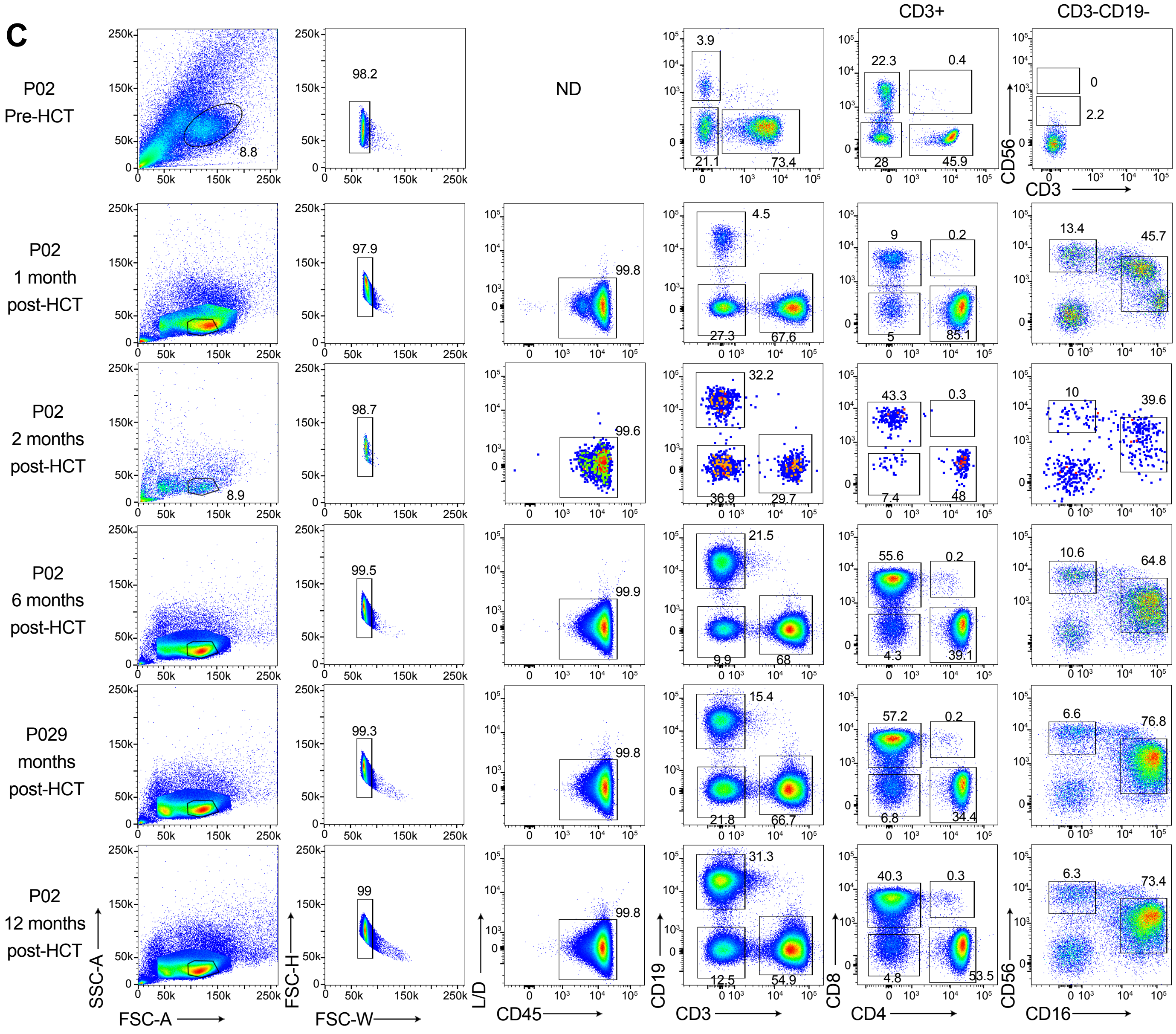

Figure S1 continued. Flow cytometric analysis and gating strategy used for identification of T cells, B cells and NK cells in peripheral blood

A-C) Flow cytometric analysis and gating strategy used for identification of T cells, B cells and NK cells in peripheral blood cryopreserved mononuclear cells from healthy donors (A), P01 (B) and P02 (C) collected at indicated timepoints. B cells are selected for by gating on viable CD45^{+} lymphocytes and are CD19^{+}, T cells are CD3+ and can be subdivided into CD4^{+}, CD8^{+} CD4^{-}CD8^{-} and CD4^{+}CD8^{+} T cells and NK cells are CD19^{-}CD3^{-} cells and are subdivided into CD56^{bright}CD16^{-} NK cells and CD56^{dim}CD16^{+} NK cells. ND = Not Determined

Supplemental figure 2

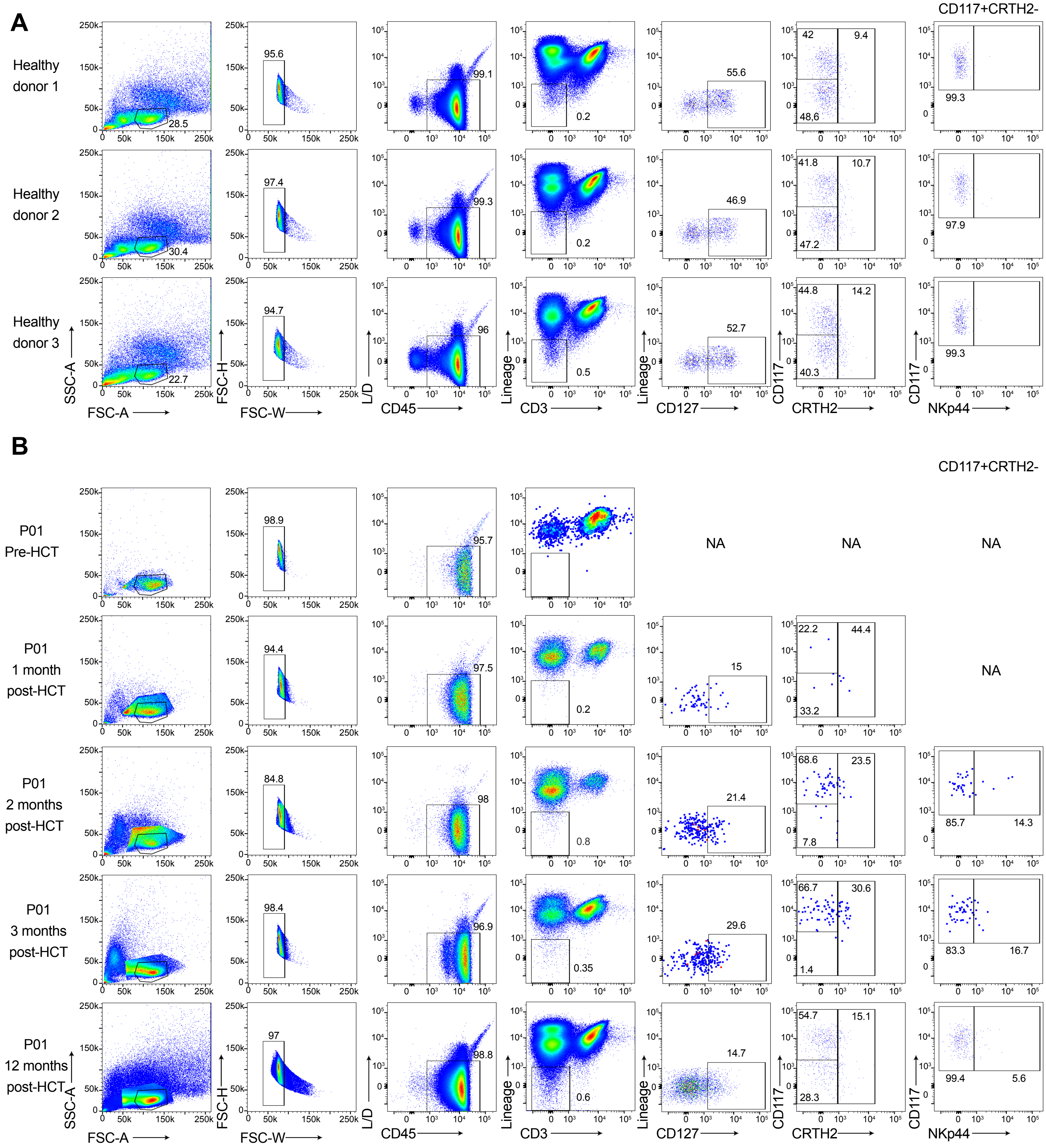

Figure S2. Flow cytometric analysis and gating strategy used for identification of ILC subsets in peripheral blood (PB) and bone marrow (BM)

Supplemental figure 2 continued

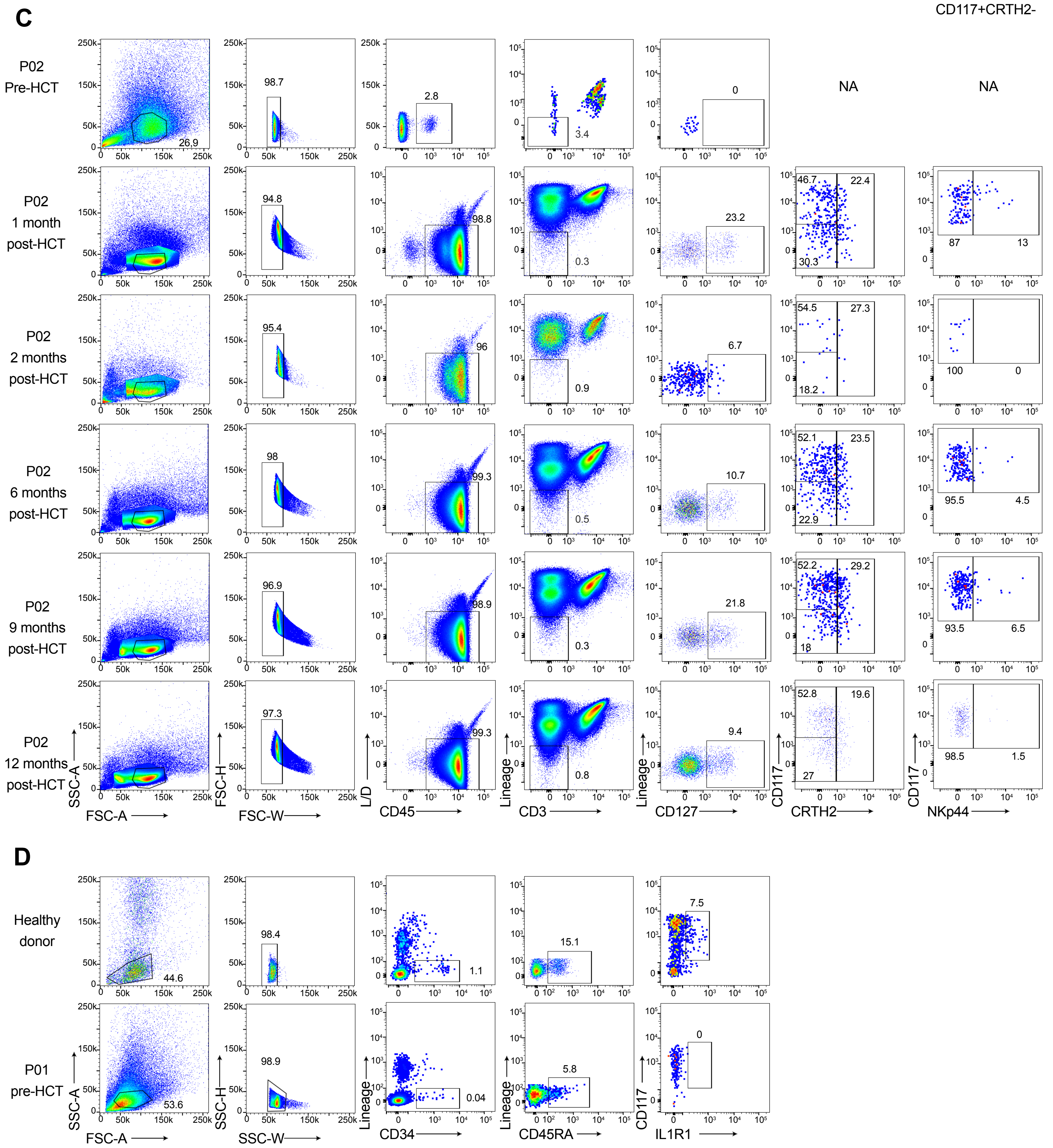

Figure S2 continued. Flow cytometric analysis and gating strategy used for identification of ILC subsets in peripheral blood (PB) and bone marrow (BM)

A-C) Flow cytometric analysis and gating strategy used for identification of ILC subsets in peripheral blood (PB) cryopreserved mononuclear cells from healthy donors (**A**), P01 (**B**) and P02 (**C**) collected at indicated timepoints. ILCs are single lymphocytes that are viable CD45+Lin-CD3-CD127+. CD117- ILC1s are gated for by selecting CD117-CRTH2- cells, ILC2s are gated for by selecting CRTH2+ cells and CD117^{+}CRTH2- cells can be subdivided into NKp44- ILCp's or NKp44^{+} ILC3. NA = Not Applicable

D) Full gating strategy used for identification of the multipotent hematopoietic progenitor cells (HPC) and BM ILC progenitors in cryopreserved mononuclear cells from BM of a healthy donor and P01. HPC are selected as CD45+Lin-CD34+CD45RA+ cells and within this population ILC precursors are selected as CD117+IL1R1+ cells. NA = Not Applicable

**Antigen (all human)**	**Clone**	**Fluorochrome**	**Company**
CD1a	HI149	FITC	Biolegend
CD3	OKT3	FITC	Biolegend
CD3	SK7	BV650	BD biosciences
CD3	UCHT1	PETX	BD biosciences
CD4	RPA-T4	FITC	Biolegend
CD4	OKT4	PETX	Biolegend
CD5	UCHT2	FITC	Biolegend
CD8	SK1	FITC	Biolegend
CD14	HCD14	FITC	Biolegend
CD16	3G8	FITC	BD biosciences
CD16	3G8	BV785	Biolegend
CD19	HIB19	FITC	Biolegend
CD19	HIB19	PE	Biolegend
CD34	581	BV421	Biolegend
CD34	581	FITC	Biolegend
CD45	HI30	AF700	Biolegend
CD45RA	HI100	APCCY7	Biolegend
CD56	HCD56	APC	Sony
CD94	18d3	FITC	Sony
CD117	104D2D1	PECy5.5	Beckman coulter
CD123	6H6	FITC	Biolegend
CD127	R34.34	PECy7	Beckman coulter
CD127	A019D5	BV421	Biolegend
CRTH2	BM16	BV421	Biolegend
CRTH2	BM16	PETX	Biolegend
BDCA2	201A	FITC	Biolegend
TCRαβ	IP26	FITC	Biolegend
TCRγδ	B1	FITC	Biolegend
FcER1α	AER-37 (CRA-1)	FITC	Biolegend
IL-1R1	Polyclonal Goat IgG	PE	R&D systems
NKp44	P44-8	AF647	Biolegend
NKp44	P44-8	PE	Biolegend
KLRG1	13F12F2	APC	eBioscience

Table S1. Antibody clones and fluorochromes used in this study
